# Recurring Functional Interactions Predict Network Architecture of Interictal and Ictal States in Neocortical Epilepsy

**DOI:** 10.1523/ENEURO.0091-16.2017

**Published:** 2017-03-08

**Authors:** Ankit N. Khambhati, Danielle S. Bassett, Brian S. Oommen, Stephanie H. Chen, Timothy H. Lucas, Kathryn A. Davis, Brian Litt

**Affiliations:** 1 Department of Bioengineering, University of Pennsylvania, Philadelphia, PA 19104; 2 Penn Center for Neuroengineering and Therapeutics, University of Pennsylvania, Philadelphia, PA 19104; 3 Department of Electrical and Systems Engineering, University of Pennsylvania, Philadelphia, PA 19104; 4 Department of Neurology, Hospital of the University of Pennsylvania, Philadelphia, PA 19104; 5 Department of Neurosurgery, Hospital of the University of Pennsylvania, Philadelphia, PA 19104

**Keywords:** dynamic network neuroscience, epileptic network, non-negative matrix factorization, functional subgraphs, prediction, interictal

## Abstract

Human epilepsy patients suffer from spontaneous seizures, which originate in brain regions that also subserve normal function. Prior studies demonstrate focal, neocortical epilepsy is associated with dysfunction, several hours before seizures. How does the epileptic network perpetuate dysfunction during baseline periods? To address this question, we developed an unsupervised machine learning technique to disentangle patterns of functional interactions between brain regions, or subgraphs, from dynamic functional networks constructed from approximately 100 h of intracranial recordings in each of 22 neocortical epilepsy patients. Using this approach, we found: (1) subgraphs from ictal (seizure) and interictal (baseline) epochs are topologically similar, (2) interictal subgraph topology and dynamics can predict brain regions that generate seizures, and (3) subgraphs undergo slower and more coordinated fluctuations during ictal epochs compared to interictal epochs. Our observations suggest that seizures mark a critical shift away from interictal states that is driven by changes in the dynamical expression of strongly interacting components of the epileptic network.

## Significance Statement

Localization-related epilepsy is a debilitating condition where seizures begin in dysfunctional brain regions and are often resistant to medication. The challenge for treating patients is mapping dysfunction in brain networks that also subserve normal function several hours before seizures. Localizing brain regions that generate seizures is critical for improving seizure freedom rates following invasive surgery. We develop new methods to identify clusters of functionally interacting brain regions from ∼100-h intracranial, neocortical recordings per epilepsy patient. Our results indicate seizure-generating brain regions: (1) can be predicted before seizures and (2) may kindle dysfunction through interactions with nonseizure generating brain regions. These findings may have clinical implications for targeting specific brain regions to control seizures several hours before they occur.

## Introduction

For ∼60 million epilepsy patients, recurring, spontaneous seizures have a crippling impact on daily life. In ∼26% of these patients, drivers of seizure activity have been linked to abnormal focal networks located in neocortical or mesial temporal structures (Siegel et al., 2001). To map dysfunction, epileptologists monitor continuous intracranial electrophysiology for biomarkers generated by the epileptic network, a set of interacting brain regions that are believed to initiate and spread seizure activity in the brain. To control seizures in medication-resistant individuals, clinical practitioners have traditionally prescribed resective surgery to remove brain tissue containing the epileptic network. More recently, epilepsy specialists are employing laser ablation and implantable devices to control dysfunction (Stacey and Litt, 2008; Fisher et al., 2010; Morrell, 2011; Tovar-Spinoza et al., 2013; Medvid et al., 2015). Novel neurotechnologies afford critical specificity in targeting brain circuits, but the key question for clinicians remains: which brain region(s) serve as the best target(s) to control a given patient’s seizures?

Localizing epileptic brain regions based on abnormal electrophysiological biomarkers is a difficult problem, as etiology, seizure semiology, and frequency of events vary greatly between patients (Kutsy et al., 1999). To reliably map the epileptic network, invasive monitoring lasts several days to weeks, and the length of the hospital stay increases the risks of infection, complications, and death. The extended monitoring period allows clinicians to describe a surgical target that accounts for variability in the seizure origin while minimizing expected impact on normal brain function. Recently, sampling error during limited monitoring time with intracranial electrodes has called into question the ability of traditional inpatient ictal recording to fully define the epileptic network (King-Stephens et al., 2015). This suggests that methods to map the epileptic network that do not rely on ictal recording may have significant advantages over current approaches. Critically, in localization-related epilepsy, brain regions that generate ictal (seizure) events are thought to be fundamentally altered in their structure and function, leading to the cognitive deficits observed during interictal (baseline) epochs (Aarts et al., 1984; Jokeit et al., 1997; Kwan and Brodie, 2001; Elger et al., 2004; Holmes and Lenck-Santini, 2006). These observations imply that brain circuits underlying cognitive functions are recruited by the epileptic network during interictal (baseline) states. However, when abnormal electrophysiology is not accompanied by discrete lesions evident on brain imaging, only ∼40% of patients attain seizure freedom following resective surgery (French, 2007). Modest outcomes associated with localization of abnormal electrophysiology suggests a fundamental gap in our understanding of how neurophysiologic biomarkers relate to pathophysiology.

A mechanistic understanding of seizure generation and evolution may be derived from spatial and temporal dynamics of the epileptic network (Wendling et al., 2003; Jerger et al., 2005; Schindler et al., 2007; Schevon et al., 2007; Schindler et al., 2008; Zaveri et al., 2009; Kramer et al., 2010; Jiruska et al., 2013; Rummel et al., 2013; Weiss et al., 2013; Burns et al., 2014; Geier et al., 2015; Khambhati et al., 2015, 2016). In this framework, network nodes are intracranial sensors measuring the electrocorticogram (ECoG) and network connections are time-varying statistical relationships between sensors (Friston, 2011; Hutchison et al., 2013). The degree of connectivity between brain regions is related to the synchronization of neural populations, a putative generator of dysfunction in epilepsy. Brain regions that are topologically central to the epileptic network tend to lie within (Wendling et al., 2003; Jerger et al., 2005; Schindler et al., 2007, 2008; Kramer et al., 2010; Jiruska et al., 2013; Burns et al., 2014; Khambhati et al., 2015) and adjacent to (Schevon et al., 2007; Zaveri et al., 2009; Rummel et al., 2013; Weiss et al., 2013; Geier et al., 2015) clinically defined seizure-onset zones (SOZs) during interictal, preictal, and ictal epochs (Zaveri et al., 2009; Warren et al., 2010; Khambhati et al., 2015). In this context, it is interesting to ask the question: if network dysfunction persists over long time scales, then (1) how does network topology drive brain dynamics differently during interictal and ictal epochs, and (2) how might aberrant brain regions disrupt functional interactions underlying normal function? Addressing these pressing questions about epileptic network physiology is crucial for targeting novel neurotechnology to dysfunctional brain circuits and minimizing impact on network structures involved in normal function.

In this work, we apply an unsupervised machine learning technique to examine how dynamic network architecture is differentially organized between ictal and interictal epochs. Our approach uncovers clusters of dynamic functional connections, or subgraphs, whose connection strengths undergo similar patterns of temporal variation, or expression, over several-day long ECoG recordings. Based on persistent network topology at the scale of ECoG (Kramer et al., 2011), we first hypothesize that meso-scale functional networks form a repertoire of subgraphs, mapping out interactions between brain regions that recur through ictal and interictal epochs. The existence of recurring subgraphs might describe fundamental connections that guide network propagation of interictal epileptiform activity in trajectories similar to seizures (Alarcon et al., 1997; Lai et al., 2007; Schevon et al., 2009; Wilke et al., 2009; Wu et al., 2010; Korzeniewska et al., 2014; Davis et al., 2016; Khambhati et al., 2016). Second, we predict that functional subgraphs pinpoint connections specific to putative regions of seizure generation from functional connectivity within interictal epochs. Third, we hypothesize that functional subgraphs undergo slower, coordinated fluctuations in ictal epochs and faster, externally driven fluctuations in interictal epochs (Khambhati et al., 2015). Our results support these hypotheses, demonstrating that functional subgraphs recur through ictal and interictal epochs, predict connectivity in the SOZ during interictal epochs, and differentiate ictal and interictal epochs on the basis of their time-varying expression.

## Materials and Methods

### Patient datasets

#### Ethics statement

All patients included in this study gave written informed consent in accordance with the Institutional Review Boards of the University of Pennsylvania and Mayo Clinic.

#### Electrophysiology recordings

Twenty-two human patients (12 male and 10 female) undergoing surgical treatment for medically refractory epilepsy believed to be of neocortical origin underwent implantation of subdural electrodes to localize the SOZ after noninvasive monitoring was indeterminate (see Table 1). De-identified patient data were retrieved from the online International Epilepsy Electrophysiology Portal (IEEG Portal) (Wagenaar et al., 2013). ECoG signals were recorded and digitized at either 512 Hz (University of Pennsylvania) or 500 Hz (Mayo Clinic) sampling rate. Surface electrode (Ad Tech Medical Instruments, Racine, WI) configurations, determined by a multidisciplinary team of neurologists and neurosurgeons, consisted of linear and two-dimensional arrays (2.3 mm diameter with 10 mm inter-contact spacing) and sampled the neocortex for epileptic foci (depth electrodes were first verified as being outside the seizure-onset zone (OUT) and subsequently discarded from this analysis). Signals were recorded using a referential montage with the reference electrode, chosen by the clinical team, distant to the site of seizure onset. Recordings spanned the duration of a patient’s stay in the epilepsy monitoring unit.

**Table 1:** Patient information

Patient (IEEG Portal)	Sex	Age (onset/surgery)	Seizure onset	Etiology	Seizure type	Ictal epochs (N)	Interictal epochs (N)	Imaging	Outcome
HUP64_phaseII	M	03/20	Left frontal	Dysplasia	CP + GTC	01	3228	L	ENGEL-I
HUP65_phaseII	M	02/36	Right temporal	Auditory reflex	CP + GTC	03	2986	N/A	ENGEL-I
HUP68_phaseII	F	15/26	Right temporal	Meningitis	CP, CP + GTC	05	3020	NL	ENGEL-I
HUP70_phaseII	M	10/32	Left perirolandic	Cryptogenic	SP	08	1079	L	NR
HUP72_phaseII	F	11/27	Bilateral left	Mesial temporal sclerosis	CP + GTC	01	2439	L	NR
HUP73_phaseII	M	11/39	Anterior right frontal	Meningitis	CP + GTC	05	1071	NL	ENGEL-I
HUP78_phaseII	M	00/54	Anterior left temporal	Traumatic injury	CP	05	1719	L	ENGEL-III
HUP79_phaseII	F	11/39	Occipital	Meningitis	CP	01	1775	L	NR
HUP86_phaseII	F	18/25	Left temporal	Cryptogenic	CP + GTC	02	2612	NL	ENGEL-II
HUP87_phaseII	M	21/24	Frontal	Meningitis	CP	02	1201	L	ENGEL-I
Study 004-2	F	14/27	Right temporal occipital	Unknown	CP + GTC	01	638	NL	ILAE-IV
Study 006	M	22/25	Left frontal	Unknown	CP	02	104	NL	NR
Study 010	F	00/13	Left frontal	Unknown	CP	02	526	L	NF
Study 011	F	10/34	Right frontal	Unknown	CP, CP + GTC	02	283	NL	NF
Study 016	F	05/36	Right temporal orbitofrontal	Unknown	CP + GTC	03	669	NL	ILAE-IV
Study 019	F	31/33	Left temporal	Unknown	CP + GTC	15	403	NL	ILAE-V
Study 020	M	05/10	Right frontal	Unknown	CP + GTC	04	412	NL	ILAE-IV
Study 023	M	01/16	Left occipital	Unknown	CP	04	208	L	ILAE-I
Study 026	M	09/09	Left frontal	Unknown	CP	10	539	NL	ILAE-I
Study 031	M	05/05	Right frontal	Unknown	CP + GTC	05	730	NL	NF
Study 033	M	00/03	Left frontal	Unknown	GA	07	1321	L	ILAE-V
Study 037	F	45/NR	Right temporal	Unknown	CP	02	1087	NL	NR

Patient datasets accessed through IEEG Portal (http://www.ieeg.org). Age at seizure onset and at electrode implant surgery are noted. Location of seizure onset (lobe) and etiology are clinically determined through medical history, imaging, and long-term invasive monitoring. Seizure types are SP (simple-partial), CP (complex-partial), CP + GTC (complex-partial with secondary generalization), or GA (generalized atonic). Counted seizures were recorded in the epilepsy monitoring unit. Interictal epochs were 5 min in duration and at least 2 h away from any seizure. Clinical imaging analysis concludes L, Lesion; NL, nonlesion. Surgical outcome is reported by both Engel score and ILAE score (scale: I--IV/V, seizure freedom to no improvement; NR, no resection; NF, no follow-up). M, male; F, female.

#### Clinical marking of the SOZ

SOZ was marked on the intracranial EEG (IEEG) according to standard clinical protocol at the University of Pennsylvania. Initial clinical markings are made on the IEEG the day of each seizure by the attending physician, always a board certified, staff epileptologist responsible for that patient’s care. Each week these IEEG markings are vetted in detail, and then finalized at surgical conference according to a consensus marking of four board-certified epileptologists. These markings on the IEEG are then related to other multimodality testing, such as brain MRI, PET scan, neuropsychological testing, ictal SPECT scanning, and magnetoenecephalographic findings to finalize surgical approach and planning. This process is standard of clinical care at National Association of Epilepsy Centers (NAEC)-certified level 4 epilepsy centers in the United States.

#### Description of ictal and interictal epochs

Ictal epochs were identified by a team of neurologists as a part of routine clinical work and spanned the period between clinically marked earliest electrographic change (EEC) (Litt et al., 2001) and termination. In this study, we disregarded subclinical seizures and only considered ictal epochs from clinical seizures that manifest seizure-related symptoms. Interictal epochs spanned 5 min in duration and were at least 2 h removed from any ictal onset. We analyzed all possible interictal epochs from patient recordings.

### Extracting time-varying functional networks

**Figure 1.**
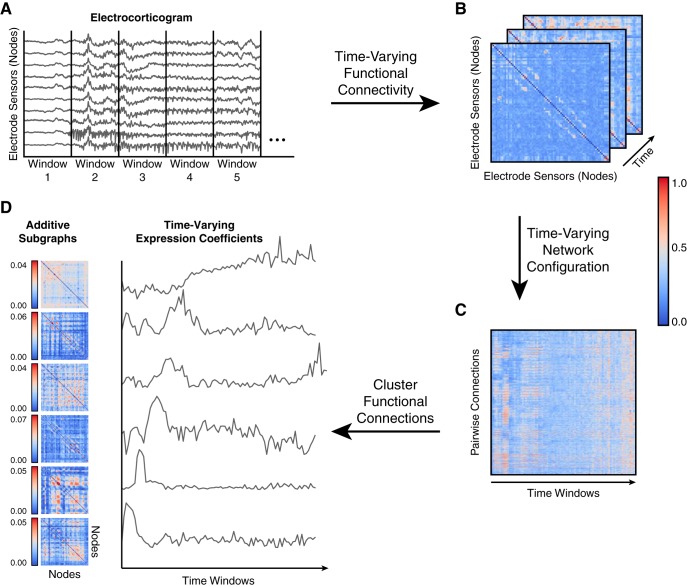
Clustering functional connections from dynamic epileptic networks. ***A***, We identify ictal and interictal epochs from ECoG signals collected from patients with drug-resistant neocortical epilepsy implanted with intracranial electrodes. An ictal epoch is the period between seizure-onset, as characterized by the EEC (Litt et al., 2001), and seizure termination. An interictal epoch is defined to be a continuous, 5-min period at least 2 h preceding or following seizure onset. To measure time-varying functional networks, we divide each epoch into 1-s time windows and estimate functional connectivity in each time window. In our model, each electrode sensor is a network node, and the weighted functional connectivity between sensors, interpreted as degree of synchrony, is represented as a network connection. ***B***, For each epoch, we estimated functional connectivity by applying a magnitude normalized cross-correlation between each pair of sensor time series in each time window. ***C***, For time-varying functional connectivity, we extract all pairwise connections between nodes and concatenate them over time windows to generate a time-varying network configuration matrix. ***D***, We apply *NMF* to the time-varying configuration matrix from each epoch, resulting in subgraphs that capture frequently repeating patterns of functional connections, and their expression over time.

Signals from each 5-min interictal epoch and each ictal epoch were divided into 1-s, nonoverlapping, stationary time windows (Fig. 1*A*) in accord with other studies (Kramer et al., 2010) and subsequently preprocessed. In each time window, signals were rereferenced to the common average reference (Towle et al., 1999; Kramer et al., 2010) to account for variation in reference location across patients and to avoid broad field effects that may erroneously bias connectivity measurements. Each window was notch filtered at 60 Hz to remove line-noise, and low-pass and high-pass filtered at 120 and 4 Hz, respectively, to account for noise, voltage drift, and *δ* frequency (0.5-4 Hz) contribution between time windows. To limit sources of volume conduction from introducing spurious connectivity, we prewhiten signals in each window using a first-order autoregressive model to account for slow dynamics. Prewhitening accomplishes two goals: (1) flattening of the signal power spectrum to enhance higher-frequency content that contains local neural population dynamics that is less affected by volume conduction, and (2) decreasing the influence of independent node dynamics when computing correlation-based connectivity measurements (Towle et al., 1999; Bullmore et al., 2001; Lund et al., 2006; Arbabshirani et al., 2014).

Time-varying functional networks were formed by applying a normalized cross-correlation similarity function ρ between the time series of two sensors in the same time window using the formula, $\rho_{xy}(k)=\underset{\tau }{\max}\left|\frac{1}{T}\sum_{t}\frac{\left(x_{k}(t)-{\bar{x}}_{k})(y_{k}(t+\tau )-{\bar{y}}_{k}\right)}{\sigma_{x_{k}}\sigma_{y_{k}}}\right|$, where **x** and **y** are signals from one of *N* sensors or network nodes, *k* is one of *K* nonoverlapping, 1-s time windows, *t* is one of *T* signal samples during the time window, τ=1,2,…,T is the time lag between signals, and ρ=0 when node *x* is the same as node *y*. The N×N×K similarity matrix is also known as a time-varying adjacency matrix **A** (Fig. 1*B*). In our weighted network analysis, we retain and analyze all possible connection weights between nodes.

An alternate representation of the three-dimensional network adjacency matrix **A** is a two-dimensional network configuration matrix $\hat{A}$, which tabulates all *N* × *N* pairwise connection strengths across *K* time windows (Fig. 1*C*). Due to symmetry of $A_{k}$, we unravel the upper triangle of $A_{k}$, resulting in the weights of N(N−1)/2 connections. Thus, $\hat{A}$ has dimensions N(N−1)/2×K. We constructed a separate network configuration matrix for each ictal and interictal epoch.

### Clustering functional connections into subgraphs

To identify network subgraphs--sets of connections whose variation in strength cluster over time--we applied an unsupervised machine learning algorithm called non-negative matrix factorization (NMF) (Lee et al., 1999) to the network configuration matrix (Fig. 1*D*). This technique enabled us to pursue a parts-based decomposition of the time-varying network configuration matrix into subgraphs with time-varying expression coefficients (Chai et al., 2017). Each subgraph is an additive component of the original network, weighted by its associated time-varying expression coefficient and represents a pattern of functional interactions between brain regions. The NMF-based subgraph learning paradigm is a basis decomposition of a collection of dynamic graphs that separates covarying network edges into subgraphs, or basis functions, with associated temporal coefficients, or basis weights. Unlike other graph clustering approaches that seek a hard partition of nodes and edges into clusters (Bassett et al., 2013), the temporal coefficients provide a soft partition of the network edges, such that the original functional network of any time window can be reconstructed through a linear combination of all the subgraphs weighted by their associated temporal coefficient of that time window (Leonardi et al., 2013, 2014; Chai et al., 2017). This implies that at a specific time window, subgraphs with a high temporal coefficient contribute their pattern of functional connections more than subgraphs with a low temporal coefficient.

Mathematically, NMF approximates $\hat{A}$ by two low-rank, non-negative matrices, such that, $\hat{A}\approx WH$, where **W** is the subgraph connectivity matrix (with dimensions N(N−1)/2×m), **H** is the time-varying expression coefficients matrix (with dimensions *m* × *K*), and *m* is the optimized number of subgraphs learned. We applied NMF to the time-varying network configuration matrix using the alternating non-negative least squares with block-pivoting method with 100 iterations for fast and efficient factorization of large matrices (Kim and Park, 2011). We initialized **W** and **H** with non-negative weights drawn from a uniform random distribution on the interval [0, 1]. Due to the nondeterministic nature of this approach, we integrated subgraph estimates over multiple runs of the algorithm using consensus clustering, a general method of testing robustness and stability of clusters over many runs of one or more nondeterministic clustering algorithms (Monti et al., 2003). Our adapted consensus clustering procedure (Greene et al., 2008; Greene, 2009) entailed the following steps: (1) run the NMF algorithm *R* times per network configuration matrix, (2) concatenate subgraph matrix **W** across *R* runs into an aggregate matrix with dimensions N(N−1)/2 × R*m, and (3) apply NMF to the aggregate matrix to determine a final set of subgraphs and expression coefficients.

In our study, we set *R* = 25 runs and separately repeated the consensus procedure for each epoch of each subject. We determined a subject-specific number of subgraphs *m* to learn across epochs by the following procedure: (1) randomly sample 50 epochs from the ictal and interictal pool, (2) apply NMF for m=2,3,…,20 subgraphs, independently for each epoch, (3) compute the Frobenius error between $\hat{A}$ and WH for each *m*, (4) retain the value for *m* that occurs at the elbow of the resulting Frobenius error curve for each patient, and (5) find the optimum number of subgraphs $\bar{m}$ as the average *m* from the 50 epochs.

In sum, this subgraph learning procedure yielded $p*\bar{m}$ total subgraphs per patient, where *p* is the total number of ictal and interictal epochs.

#### Generating surrogate subgraphs

An important mathematical property of subgraphs is that they form a basis set of the time-varying functional network from which they were derived. In other words, there exists a linear combination of an epoch’s subgraphs that reconstruct the original network, and any linear combination of the subgraphs forms a new subgraph that is still a basis of the original network. These properties allowed us to construct surrogate subgraphs with rewired network topology that maintain their basis functionality and preserve the empirically observed distribution of connection strengths.

**Figure 2.**
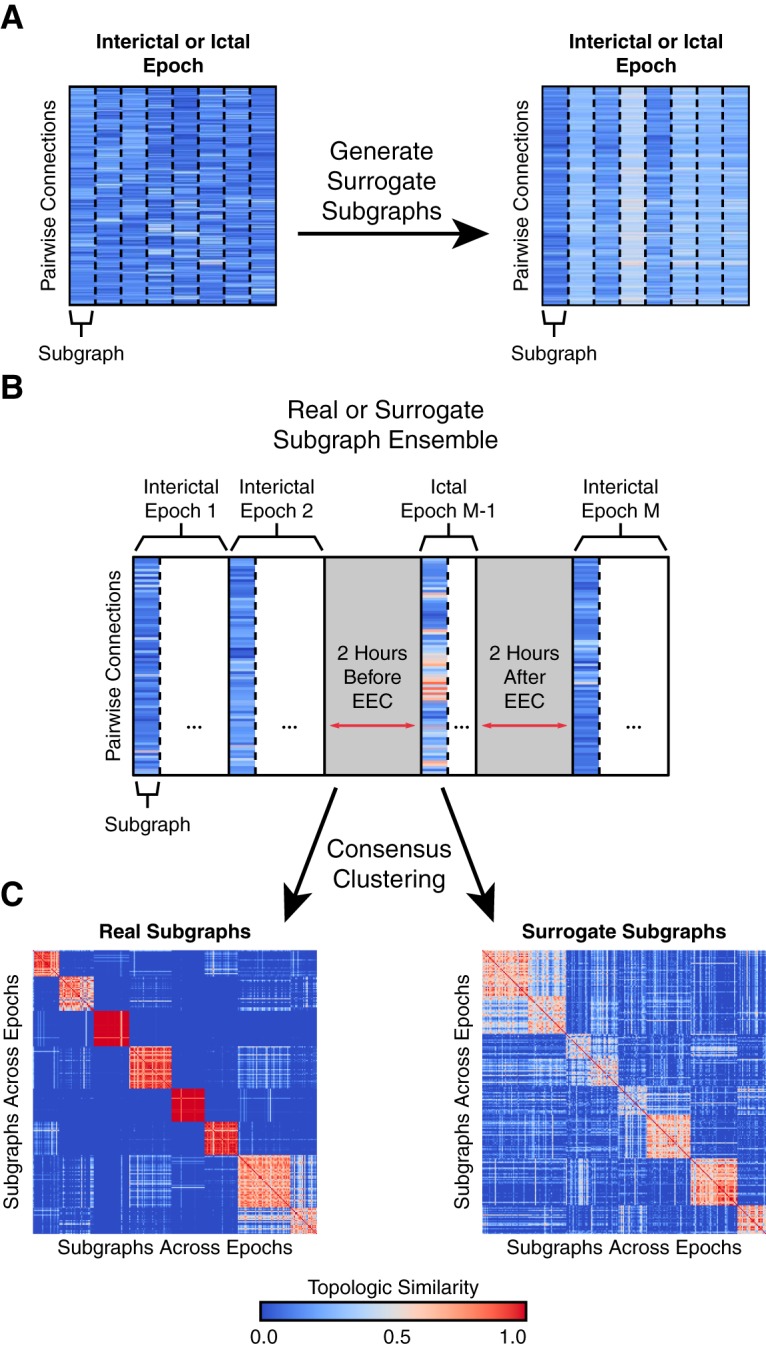
Clustering subgraphs based on topological similarity. ***A***, For the set of original subgraphs learned from an epoch of data (left), we generated an equally sized set of surrogate subgraphs (right) by computing a weighted linear combination of the subgraphs with weights drawn from a uniform random distribution on the interval [0, 1]. The surrogate subgraphs have rewired network topology but maintain their functionality as a mathematical basis of the original network. ***B***, For each patient, we constructed a subgraph ensemble matrix, representing the N(N−1)/2 functional connections for each subgraph from all interictal and ictal epochs. The ensemble matrix aggregates functional subgraphs expressed over ∼100 h of intracranial recording. We also constructed a patient-specific surrogate ensemble matrix by aggregating surrogate subgraphs across all epochs. ***C***, We quantified the topological similarity between all subgraphs in the ensemble matrix by applying a consensus NMF algorithm that tracks the number of times every pair of subgraphs is assigned to the same cluster over 100 runs of NMF. This procedure resulted in a coclustering probability matrix representing the frequency with which subgraphs from ictal and interictal epochs are clustered together, a measure of similarity between the connectivity profiles of subgraph pairs. In the example, the coclustering probability matrix of real subgraphs demonstrates less ambiguous similarity (matrix entries are near 0 or 1) and greater clustering than surrogate subgraphs (matrix entries closer to 0.5).

We formed a set of surrogate subgraphs for each epoch by calculating a linear combination of the original subgraphs with weights pooled from a uniform random distribution on the interval [0, 1] (Fig. 2*A*). The size of the surrogate subgraph set remained equal to the size of the original subgraph set.

### Clustering subgraph ensembles across epochs

We sought to describe subgraph topology from the entirety of a patient’s data record by quantifying the similarity of subgraph connectivity profiles between interictal and ictal epochs. While several similarity and distance metrics are capable of comparing statistical features across observations in a dataset (e.g., Pearson’s correlation, Euclidean distance, cosine similarity), recent work has shown that a probabilistic measure of similarity derived from consensus clustering, by leveraging the nondeterministic property of the random initialization, may more accurately identify clusters in large datasets with many features (Monti et al., 2003). To quantify topological similarity of subgraphs across all of a patient’s epochs, we again employed an NMF-based consensus clustering approach.

First, we compiled subgraphs across all of a patient’s epochs and constructed a subgraph ensemble matrix **E** (with dimensions $N(N-1)/2\times (p*\bar{m})$; Fig. 2*B*). To cluster the collection of $p*\bar{m}$ subgraphs, we applied multiple runs of NMF to **E**, such that E≈VG, where **V** represents the subgraph for each cluster centroid (with dimensions N(N−1)/2×j) and **G** represents the likelihood cluster assignment for each subgraph (with dimensions $j\times (p*\bar{m})$), where *j* is the number of patient-wide clusters of subgraphs. After every NMF run, we retrieved the cluster assignment with maximum likelihood for each subgraph and counted the number of times each possible pair of subgraphs was assigned to the same cluster, and by extension the probability that any two subgraphs cocluster (Brunet et al., 2004; Greene et al., 2008; Greene, 2009). These probabilities were tabulated in a symmetric coclustering probability matrix **S** (with dimensions $\left(p*\bar{m})\times (p*\bar{m}\right)$; Fig. 2*C*).

For every patient, we computed a coclustering probability matrix **S** over 100 NMF runs for each number of subgraph clusters j=2,3,…,20. To determine the optimum number of clusters *j*, we computed the Frobenius error between **E** and **VG** for each *j* and retained the value $\bar{j}$ that occurs at the elbow of the resulting Frobenius error curve for each patient. Finally, we assigned each subgraph to its consensus cluster by applying one run of NMF, with $\bar{j}$ clusters, to **S**.

To generate a surrogate coclustering probability matrix, we repeated our approach and replaced the original subgraphs in **E** with surrogate subgraphs and set the number of subgraphs *j* to the optimized number of subgraphs $\bar{j}$ from the original ensemble clustering.

#### Two-dimensional projection of subgraph similarity

**Figure 3.**
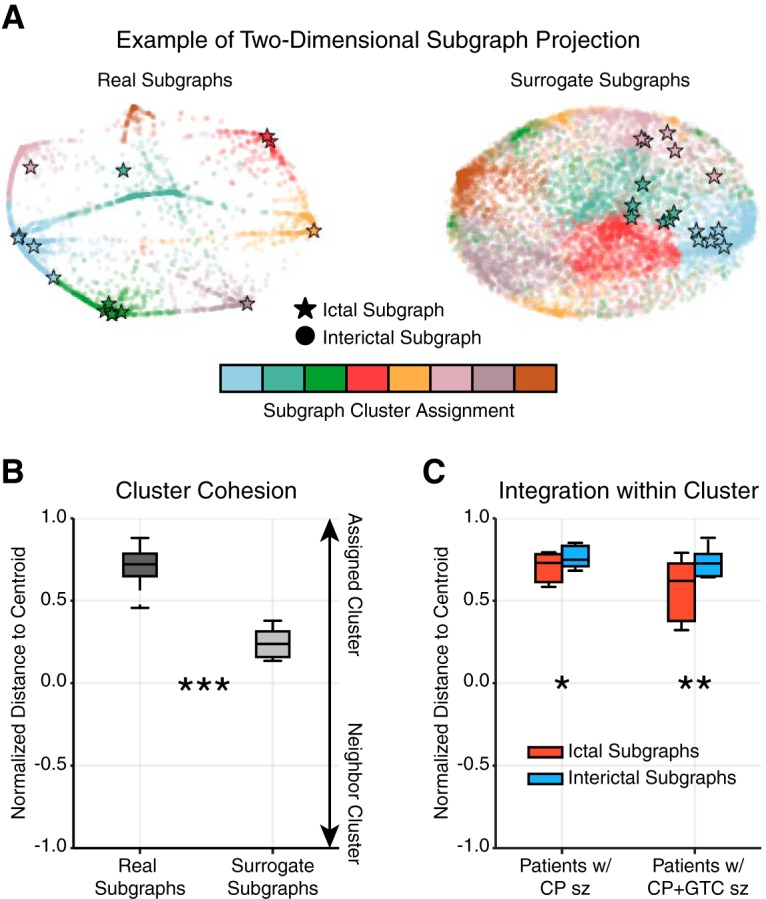
Ictal subgraphs are recapitulated during interictal epochs. ***A***, Example two-dimensional projection of a patient’s subgraph coclustering probability matrix. Each marker represents a subgraph from a single epoch and the distance between a subgraph pair indicates their topological similarity (i.e., closer subgraphs are more similar); circles represent interictal subgraphs and bolded stars represent ictal subgraphs; colors represent cluster assignment based on consensus clustering of the subgraph ensemble. The projections of real subgraphs (left) of the same cluster (color) tend to be closer to one another than to subgraphs of other clusters. In contrast, the projections of surrogate subgraphs from the same cluster tend to be as close to one another as surrogate subgraphs from other clusters. ***B***, Normalized, projected distance of a subgraph to its assigned cluster’s centroid, the mean geographical location of subgraphs in a cluster, relative to its neighboring cluster’s centroid (most proximal, nonassigned cluster centroid), averaged over all subgraphs of each patient (*N* = 22). Real subgraphs were significantly closer to their cluster centroid compared with surrogate subgraphs (paired $t$-test; *t*_21_ = 12.09, *p* < 7 × 10^−11^), suggesting the same set of brain regions functionally interact repeatedly over several hours. ***C***, Normalized, projected distance of ictal and interictal subgraphs to their cluster centroid, averaged over all ictal or interictal subgraphs of each patient with complex partial (CP) seizures (*N* = 8) and with secondarily generalized complex partial (CP + GTC) seizures (*N* = 10). Both groups of patients expressed ictal subgraphs that were significantly further away from their cluster centroid than interictal subgraphs (paired $t$-test; CP: *t*_7_ = −3.29, *p* = 0.013; CP + GTC: *t*_9_ = −4.26, *p* = 0.002), suggesting ictal subgraphs may represent functional connections that lie at the transition between interictal subgraphs. (*p < 0.05, **p < 0.01, ***p < 0.001; Bonferroni corrected for multiple comparisons).

To study the overall topological similarity between subgraphs, we employed a multidimensional scaling method (Borg and Groenen, 2005) that projects each of the $p\times \bar{m}$ subgraphs as a data point in two-dimensional space and constrains the position of each data point a distance away from all other data points based on their relative similarities, as specified in **S**. In other words, more topologically similar (dissimilar) subgraphs are closer (further) in two-dimensional space (for example, see Figure 3*A*). Formally, MDS assigns each subgraph a two-dimensional coordinate (*xy*) by minimizing the following stress function, ${\text{Stress}}_{S}={\left(\sum_{i\neq j=1,\ldots ,p*\bar{m}}{\left(1-S_{ij}-\left||xy_{i}-xy_{j}\right||\right)}^{2}\right)}^{1/2}$, where **S** is the probabilistic subgraph coclustering matrix, *i* and *j* are each different indices for one of $\bar{m}$ subgraphs of the *p* epochs. The MDS procedure assigns each subgraph a two-dimensional *xy* coordinate.

Using the two-dimensional subgraph projection, we studied the proximity of a subgraph to its cluster centroid. Subgraphs closer to the centroid of their assigned cluster were considered more integrated, while subgraphs closer to the centroid of a nonassigned cluster (neighboring cluster) were considered more promiscuous. Formally, we computed a normalized distance to centroid measure by, $\text{Distance}(p,m,j_{\text{assign}},j_{\text{neighbor}})=\frac{\text{Dist}[xy_{p,m},{\bar{xy}}_{j_{\text{neighbor}}}]-\text{Dist}[xy_{p,m},{\bar{xy}}_{j_{\text{assign}}}]}{\text{Dist}[xy_{p,m},{\bar{xy}}_{j_{\text{neighbor}}}]+\text{Dist}[xy_{p,m},{\bar{xy}}_{j_{\text{assign}}}]}$, where Dist is the Euclidean distance function, *xy* are projected coordinates of the $m^{\text{th}}$ subgraph of the $p^{\text{th}}$ epoch, and $\bar{xy}$ is the centroid coordinate of the assigned cluster for the subgraph $j_{\text{assign}}$ or the centroid coordinate of the most proximal, nonassigned cluster $j_{\text{neighbor}}$. Intuitively, a subgraph closer to the centroid of its assigned cluster than its neighboring cluster has normalized distance near +1, a subgraph closer to the centroid of its neighboring cluster than its assigned cluster has normalized distance near –1, and a subgraph equally distant to its own cluster centroid and neighboring cluster centroid has normalized distance of 0 (for example, see Figure 3*B*,*C*).

### Measures of subgraph topology and dynamics

To quantify the topological and dynamic role of functional subgraphs in the epileptic network, we describe several measures based on the distributions of subgraph connectivity and expression coefficients.

**Figure 4.**
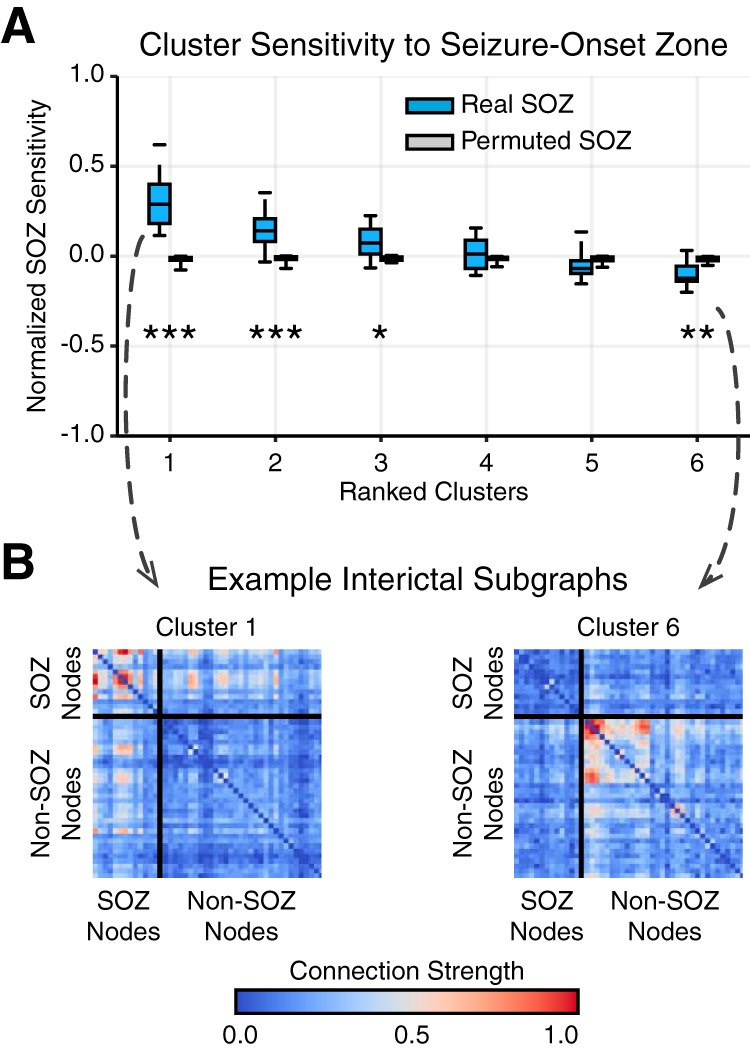
Interictal subgraphs are selectively sensitive to the SOZ. ***A***, Distribution of average SOZ sensitivity of subgraphs in each cluster, ranked in decreasing order, from each patient (*N* = 22). SOZ sensitivity of true SOZ labels in blue and of permuted SOZ labels in gray. We observed a significant effect of SOZ sensitivity for real SOZ labels compared with permuted SOZ labels for clusters 1, 2, 3, and 6 (*p < 0.05, **p < 0.01, ***p < 0.001; Bonferroni corrected for multiple comparisons). These results demonstrate that functional interactions between brain regions are heterogeneously sensitive to dysfunction in the SOZ, depending on cluster-specific subgraph stereotypes. ***B***, Importantly, we observed that subgraphs of cluster 1 were significantly sensitive to connections within the SOZ, while subgraphs of cluster 6 were significantly sensitive to connections outside the SOZ. An example of subgraphs from cluster 1 (left) and cluster 6 (right) are shown here. Connections between SOZ nodes are shown in the top-left box, and connections between non-SOZ nodes are shown in the bottom-right box.

To determine the degree to which a subgraph expressed functional connectivity in the SOZ, we computed SOZ sensitivity, a measure of the relative strength of subgraph connectivity within the seizure-onset zone (SOZ) and OUT. Mathematically, the SOZ sensitivity is defined, SOZ Sensitivity $(p,m)=\frac{{\bar{C}}_{p,m_{\text{SOZ}}}-{\bar{C}}_{p,m_{\text{OUT}}}}{{\bar{C}}_{p,m_{\text{SOZ}}}+{\bar{C}}_{p,m_{\text{OUT}}}}$, where ${\bar{C}}_{\text{SOZ}}$ is the average subgraph connection strength of nodes within the SOZ and ${\bar{C}}_{\text{OUT}}$ is the average subgraph connection strength of nodes outside the SOZ, of the $m^{\text{th}}$ subgraph of the $p^{\text{th}}$ epoch. The SOZ sensitivity ranges from +1, maximally sensitive to functional connections within the SOZ, to –1, maximally sensitive to functional connections outside the SOZ (for example, see Figure 4). We also computed a surrogate distribution of SOZ sensitivity by randomly permuting the SOZ label across network nodes and recomputing SOZ sensitivity.

To determine the degree to which a subgraph expressed functional connectivity between brain regions exhibiting intericital epileptic spikes, we computed a spike sensitivity measure of the relative strength of subgraph connectivity within spiking regions and outside spiking regions. Mathematically, the spike sensitivity is defined as Spike Sensitivity $(p,m)=\frac{{\bar{C}}_{p,m_{\text{spike}}}-{\bar{C}}_{p,m_{\text{nonspike}}}}{{\bar{C}}_{p,m_{\text{spike}}}+{\bar{C}}_{p,m_{\text{nonspike}}}}$, where ${\bar{C}}_{\text{spike}}$ is the average subgraph connection strength of nodes within spiking regions and ${\bar{C}}_{\text{nonspike}}$ is the average subgraph connection strength of nodes outside spiking regions of the $m^{\text{th}}$ subgraph of the $p^{\text{th}}$ epoch. The spike sensitivity ranges from +1 (maximally sensitive to functional connections within spiking regions) to –1 (maximally sensitive to functional connections outside spiking regions). We also computed a surrogate distribution of spike sensitivity by randomly permuting the spike label across network nodes and recomputing spike sensitivity.

**Figure 5.**
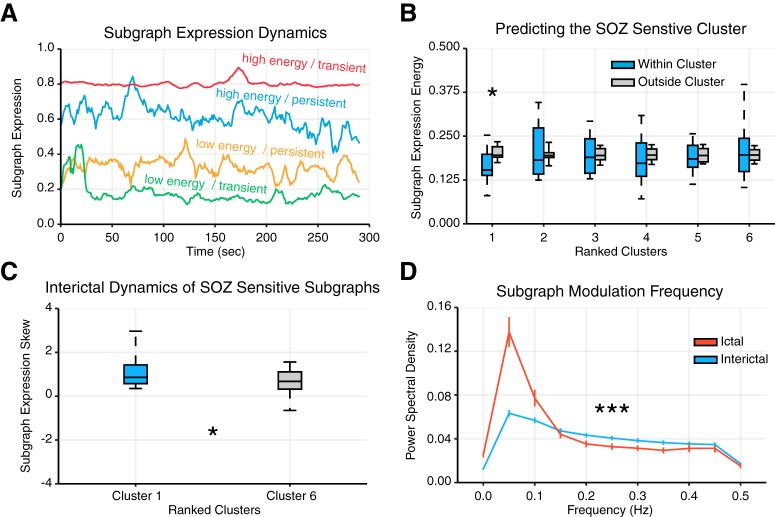
Expression energy and transience differentiate ictal and interictal epochs. ***A***, We computed (i) subgraph expression energy--the overall activity of a subgraph--and (ii) subgraph expression skew--the temporal transience or persistence of a subgraph’s activity. Shown here are four examples of subgraph expression from a single patient, chosen by identifying subgraphs whose expression energy and expression skew were in the bottom and top third of all epochs. These examples demonstrate high energy and transience (red), high energy and persistence (blue), low energy and persistence (yellow), and low energy and transience (green). ***B***, Distribution of subgraph expression energy, averaged across interictal epochs of each cluster (ranked by SOZ sensitivity) for each patient (*N* = 22). For each cluster, we compared the distribution of expression energy for subgraphs of that cluster to expression energy for subgraphs of all other clusters and found significantly lower expression energy of subgraphs within cluster 1, most sensitive to nodes in the SOZ, than outside cluster 1 (paired $t$-test; *t*_21_ = −3.21, *p* = 0.004; Bonferroni corrected for multiple comparisons). ***C***, Distribution of subgraph expression skew, averaged across interictal epochs of clusters 1 and 6 for each patient (*N* = 22). We observed subgraphs of cluster 1, which were most sensitive to nodes in the SOZ, exhibited significantly greater skew, and therefore greater temporal transience, than subgraphs of cluster 6, which were most sensitive to nodes outside the SOZ (paired *t* test; *t*_21_ = 2.12, *p* = 0.04). These findings suggest that subgraphs with strongly connected SOZ nodes exhibit more transient, burst-like, dynamics than subgraphs with strongly connected non-SOZ nodes. ***D***, PSD distribution of ictal and interictal subgraph expression, averaged over patients (*N* = 22). We observed a significant difference between ictal and interictal subgraph expression, ictal subgraphs modulate their expression at lower frequencies and interictal subgraphs modulate their expression at higher frequencies (FDA; $p<3\times 10^{-5}$). These findings suggest that subgraph expression is more gradual and coordinated during ictal epochs than interictal epochs.

We compared subgraph dynamics between epochs by calculating the energy, skew, and power spectral density (PSD) of subgraph expression coefficients. To compare subgraph expression between different epochs, we normalized each subgraph’s expression coefficients such that its maximum value was 1. The subgraph expression energy (Chai et al., 2017) quantifies the overall magnitude expression of the subgraph during an epoch (for example, see Figure 5*C*) and was computed by $\text{energy}(p,m)=E[H_{p,m}{}^{2}]$, where **H** are the temporal coefficients of the $m^{\text{th}}$ subgraph from the $p^{\text{th}}$ epoch.

The skew of a distribution of subgraph expression coefficients quantifies how transiently or persistently subgraphs are expressed (Chai et al., 2017). Intuitively, transient subgraphs are expressed in brief, infrequent bursts, resulting in a heavy-tailed distribution of temporal coefficients (i.e., more small coefficients and few large coefficients), and persistent subgraphs are expressed evenly in time, resulting in a more normal distribution of temporal coefficients that fluctuate about the mean. The skew of the distribution of temporal coefficients for a subgraph distinguishes whether it is transiently (skew is greater than zero) or persistently (skew less than zero) expressed (for example, see Figure 5*D*). The skew of the subgraph expression coefficients during an epoch is defined as $\text{skew}(p,m)=\frac{E[{\left(H_{p,m}-\mu_{H_{p,m}}\right)}^{3}]}{{\left(E[{\left(H_{p,m}-\mu_{H_{p,m}}\right)}^{2}]\right)}^{3/2}}$, where **H** are the temporal coefficients of the $m^{\text{th}}$ subgraph from the $p^{\text{th}}$ epoch, and *μ_H_* is the mean of the coefficients.

The PSD quantifies the modulation frequency of a subgraph’s expression (Leonardi et al., 2013) during an epoch and was computed using Welch’s method with a sampling frequency of 1 Hz (corresponding to the duration of ECoG signal used to measure functional connectivity) and an FFT window size of 20 s (for example, see Figure 5*D*).

### Statistical tests

We performed statistical tests at the patient level for each analysis in this study by first averaging measures across all subgraphs or subgraph clusters for a given patient, and measuring effects over all patients.

First, we examined the topological similarity between ictal, interictal, and surrogate subgraphs within patients. To compare each of these subgraph types, we calculated the average distance to centroid for all subgraphs of each type and used paired $t$-tests to examine differences within patients (Table 2, a--c).

**Table 2:** Statistical table

Line	Data structure	Type of test	Power
a	Normal	Paired $t$-test	1
b	Normal	Paired $t$-test	0.21
c	Normal	Paired $t$-test	0.57
d	Normal	Paired $t$-test (Bonferroni corrected)	1
e	Normal	Paired $t$-test (Bonferroni corrected)	0.99
f	Normal	Paired $t$-test (Bonferroni corrected)	0.97
g	Normal	Paired $t$-test (Bonferroni corrected)	0.85
h	Normal	Paired $t$-test (Bonferroni corrected)	0.10
i	Normal	Paired $t$-test (Bonferroni corrected)	0.10
j	Normal	Paired $t$-test (Bonferroni corrected)	0.55
k	Normal	Paired $t$-test	0.58
l	Nonparametric	Permutation test	$1\times 10^{6}$ permutes
m	Normal	One-way ANOVA	1
n	Normal	Paired $t$-test (Bonferroni corrected)	0.70
o	Normal	Paired $t$-test (Bonferroni corrected)	0.36
p	Normal	Paired $t$-test (Bonferroni corrected)	0.06
q	Normal	Paired $t$-test (Bonferroni corrected)	0.07
r	Normal	Paired $t$-test (Bonferroni corrected)	0.05
s	Normal	Paired $t$-test (Bonferroni corrected)	0.05
t	Nonparametric	Permutation test	$1\times 10^{6}$ permutes

Next, we assessed whether certain clusters of subgraphs were more sensitive to functional connectivity in the SOZ than others. Using a paired $t$-test and Bonferroni multiple comparisons correction, we compared the SOZ sensitivity distribution of each cluster to a null model in which brain regions within the SOZ are randomly permuted for every interictal subgraph (Table 2, d--i). We similarly assessed whether certain clusters of subgraphs were more sensitive to functional connectivity in interictal spiking regions than others. Using a paired $t$-test and Bonferroni multiple comparisons correction, we compared the spike sensitivity distribution of each cluster to a null model in which brain regions exhibiting interictal spikes are randomly permuted for every interictal subgraph (Table 2, n--s). To determine whether a significant effect between subgraph cluster assignment and spike sensitivity exists, we used a one-way ANOVA (Table 2, m).

Next, we investigated whether subgraphs of different clusters exhibit different degree of expression energy during interictal epochs. Using a paired $t$-test and Bonferroni multiple comparisons correction, we compared the distribution of expression energy, averaged over all interictal subgraph of each cluster, across patients, to the distribution of expression energy, averaged over all interictal subgraphs outside that cluster, across patients (Table 2, j). Similarly, using a paired $t$-test, we compared the distribution of expression skew between subgraphs of clusters with high SOZ sensitivity and subgraphs of clusters with low SOZ sensitivity (Table 2, k).

We next compared the average PSD curves between ictal and interictal epochs using a statistical technique called functional data analysis (FDA; see Ramsay and Silverman, 2005, for technique, and Bassett et al., 2012, for illustrative application). FDA allowed us to test whether the area between ictal and interictal PSD curves were significantly different by comparing the true area to a null model in which ictal and interictal labels across subjects were permuted 1,000,000 times and the area between the curves was recomputed for each permutation (Table 2, l and t).

## Results

To disentangle functional subgraphs and their time-varying expression from epileptic brain, we retrieved ECoG recorded during ictal and interictal epochs from 22 patients undergoing routine presurgical evaluation of their neocortical epilepsy (see Table 1 for patient-specific information) through the IEEG Portal (http://www.ieeg.org). We defined an ictal epoch as the period of ECoG signal between seizure onset, as characterized by the EEC (Litt et al., 2001), and seizure termination. Further, we defined an interictal epoch as a continuous 5-min period of ECoG signal at least 2 h preceding or following seizure onset. We analyzed all possible interictal epochs, which amounted to μ=106±17 h of ECoG signal per patient.

For each epoch of each patient, we applied the following steps: (1) estimated weighted functional connectivity using a normalized cross-correlation metric and (2) clustered patterns of frequently expressed functional connections from the network model by applying a machine learning technique called NMF to the time-varying network configuration matrix (see Materials and Methods for detailed procedure, and see Table 3 for number of subgraphs learned per epoch for each patient). This technique enabled us to pursue a parts-based decomposition of functional connections into subgraphs with time-varying expression coefficients (Chai et al., 2017). Each subgraph is an additive component of the original network and represents a pattern of functional interactions between brain regions. Subgraphs are accompanied by time-varying expression coefficients, measuring the degree to which each subgraph is expressed at a given point in time.

**Table 3:** Subgraph learning and ensemble clustering table

Patient (IEEG Portal)	Electrode sensors (*N*)	Electrode configuration	Ictal Epochs (N)	Interictal Epochs (N)	Total Epochs (p)	Subgraphs per Epoch ($\bar{m}$)	Subgraph Ensemble Clusters ($\bar{j}$)
HUP64_phaseII	88	Grid: 8x8; Strip: 1x6 (4)	01	3228	3229	8	8
HUP65_phaseII	80	Grid: 8x8; Strip: 1x6 (3)	03	2986	2989	8	9
HUP68_phaseII	79	Grid: 8x8; Strip: 1x8 (2), 1x4 (2)	05	3020	3025	8	7
HUP70_phaseII	78	Grid: 8x8; Strip: 1x6, 1x4 (2)	08	1079	1087	7	8
HUP72_phaseII	62	Strip: 1x8 (3), 1x6 (5), 1x4 (2)	01	2439	2440	8	9
HUP73_phaseII	56	Strip: 1x8 (4), 1x6 (4)	05	1071	1076	8	7
HUP78_phaseII	100	Grid: 8x8; Strip: 1x6 (2), 1x4 (3); Depth: 1x4 (3)	05	1719	1724	6	8
HUP79_phaseII	84	Grid: 6x8; Strip: 1x8, 1x6 (4), 1x4	01	1775	1776	8	8
HUP86_phaseII	118	Grid: 8x8; Strip: 1x6 (5), 1x4 (4); Depth: 1x4 (2)	02	2612	2614	7	8
HUP87_phaseII	88	Grid: 8x8; Strip: 1x4 (3); Depth: 1x4 (3)	02	1201	1203	8	8
Study 004-2	64	Grid: 6x6; Strip: 1x4 (5); Depth: 1x4 (2)	01	638	639	8	7
Study 006	56	Grid: 6x8; Strip: 1x8	02	104	106	8	8
Study 010	56	Grid: 6x8; Strip: 1x4 (2)	02	526	528	8	10
Study 011	84	Grid: 6x8; Strip: 1x8 (2), 1x4 (5)	02	283	285	7	7
Study 016	64	Grid: 4x6 (2); Strip: 1x4 (4)	03	669	672	8	6
Study 019	80	Grid: 3x8, 6x6; Strip: 1x8 (2), 1x4 (3); Depth: 1x4 (2)	15	403	418	7	8
Study 020	56	Grid: 4x4, 4x6; Strip: 1x4 (4)	04	412	416	8	9
Study 023	92	Grid: 8x8; Strip: 1x8, 1x4 (3); Depth: 1x4 (2)	04	208	212	8	8
Study 026	96	Grid: 8x8; Strip: 1x8 (3), 1x4 (2)	10	539	549	7	6
Study 031	116	Grid: 8x8, 4x6; Strip: 1x8 (2), 1x4 (3)	05	730	735	7	7
Study 033	124	Grid: 8x8, 3x8; Strip: 1x8 (3), 1x4 (3)	07	1321	1328	8	7
Study 037	80	Grid: 8x8; Strip: 1x8 (2)	02	1087	1089	8	9

Summary of number of ictal and interictal epochs, total number of epochs, optimized number of subgraphs learned per epoch, and optimized number of subgraph ensemble clusters for each patient.

Importantly, our approach yields a collection of functional subgraphs over the long-term clinical recording. We studied the topology and dynamics of these learned subgraphs in greater detail to understand and pinpoint drivers of epileptic network dysfunction, interictally.

### Ictal network architecture emerges during interictal epochs

We first ask the following: do subgraphs of interacting brain regions recur in their expression over the entire duration of a patient’s intracranial recordings? We expected that if the same brain regions interact frequently, as described by a subgraph, then similar patterns of subgraph connectivity should emerge over the long-term recording. To test our hypothesis, we took the following probabilistic approach (Fig. 2): (1) constructed a subgraph ensemble matrix by aggregating functional connections over all subgraphs of a patient, (2) quantified topological similarity between subgraphs by applying a consensus NMF algorithm to separate ensemble matrices for real and surrogate subgraphs, (3) populated a real and a surrogate coclustering probability matrix based on pairwise similarity of subgraphs from all epochs, and (4) projected the coclustering probability matrix on a two-dimensional Euclidean space using MDS. See Table 3 for number of subgraph ensemble clusters for each patient.

In the two-dimensional projection space, topologically similar subgraphs are geographically closer and topologically dissimilar subgraphs are geographically farther from one another. We expected that interactions between brain regions prescribed by subgraphs within a cluster would be highly distinct from interactions between brain regions of other clusters. We visually confirmed this hypothesis in a sample patient, observing that geographically closer subgraphs were more likely assigned to the same cluster (Fig. 3*A*). In contrast, surrogate subgraphs, with randomized connectivity, of the same patient did not exhibit geographical clustering corresponding to the clustering assignment. To test whether clustering of topologically similar subgraphs is significantly greater in the true data than in the surrogate model, we quantified the degree of clustering by computing a normalized distance to centroid index for each subgraph that compares the Euclidean distance from a subgraph to its assigned cluster’s centroid and the same subgraph to its nearest neighboring cluster centroid (Fig. 3*B*). A cluster centroid is the mean two-dimensional, geographical location over all subgraphs in the cluster. Using a paired $t$-test, we found that the normalized distance to centroid, averaged over all subgraphs for each patient, was significantly greater for real subgraphs (μ=0.71±0.03) than surrogate subgraphs (μ=0.24±0.02; $t_{21}=12.09,\;p<7\times 10^{-11}$; Table 2, a). These results suggest that subgraphs assigned to the same cluster exhibit greater topological similarity than expected by chance. In other words, the functional architecture of meso-scale brain circuits is organized by recurring subgraphs of connectivity, in which the same sets of brain regions functionally interact, repeatedly, over several hours. These recurring patterns of functional interactions describe organizational rules for specific groups of brain regions more likely to functionally interact at different periods of time.

Based on our result of recurring functional subgraphs in epileptic brain, we next asked whether ictal subgraphs are topologically distinct from interictal subgraphs. Visualizing the two-dimensional projection of the subgraph coclustering probability matrix from an example patient (Fig. 3*A*), we observed several bridge-like extensions between subgraph clusters, representing putative transition subgraphs between clusters that might be invoked as the network shifts between dynamical states. We hypothesized that ictal subgraphs lie closer to the cluster periphery, at the junction of subgraph transitions, than interictal subgraphs. Moreover, we expected subgraphs of seizures that undergo more complex stages of spreading dynamics, secondarily generalized, complex partial seizures (CP + GTC), would be closer to these junctions (i.e., further from the cluster centroid) than focal seizures whose dynamics minimally spread, complex partial seizures (CP). To test our hypothesis, we computed the normalized distance to centroid index, separately, for ictal and interictal subgraphs of each patient with CP seizures and with CP + GTC seizures (Fig. 3*C*). Using a paired $t$-test and Bonferroni correction for multiple comparisons, we found: (1) for patients with CP seizures, ictal subgraphs were significantly more distant (μ=0.70±0.04) from their cluster centroid than interictal subgraphs (μ=0.76±0.03; $t_{7}=-3.29$, *p* = 0.013; Table 2, b); and (2) for patients with CP + GTC seizures, ictal subgraphs were significantly more distant (μ=0.57±0.06) from their cluster centroid than interictal subgraphs (μ=0.71±0.04; $t_{9}=-4.26$, *p* = 0.002; Table 2, c). These results suggest that ictal subgraphs are less integrated within their clusters than interictal subgraphs and that ictal subgraphs of patients with CP + GTC seizures ($t_{9}=-4.26$) lie further from cluster centroid than ictal subgraphs of patients with CP seizures ($t_{7}=-3.29$). Importantly, ictal subgraphs are not topologically distinct from interictal subgraphs and may, in fact, represent functional connections that lie at the transition between interictal subgraphs. Furthermore, seizures with complex patterns of spreading dynamics (CP + GTC) may express functional connections closer to junctions between subgraph clusters than seizures with more focal dynamics (CP).

### Interictal subgraphs predict seizure-onset regions

In the preceding analyses, we observed that (1) ictal and interictal subgraphs that are more topologically similar are grouped in the same cluster and (2) ictal subgraphs are topologically similar to interictal subgraphs and may capture transitions between clusters. If similar patterns of functional connectivity are expressed during ictal and interictal epochs, then we logically ask whether interictal subgraphs can predict which functional interactions drive seizure-onset. To address this question, we compared interictal subgraph topology within and outside of clinically defined seizure-onset brain regions. In accord with routine clinical evaluation of patients’ epilepsy, a team of neurologists successfully identified the sensors in the SOZ and sensors exhibiting interictal epileptiform spikes based on visual inspection of the intracranial recordings.

To determine the degree to which a subgraph expressed functional connectivity in the SOZ, we quantified the relative strength of brain regions within the SOZ and OUT for each subgraph by computing the SOZ sensitivity measure. This measure enabled us to summarize the relationship between functional subgraphs and the SOZ of each patient and compare subgraph architecture between patients.

We first asked whether all interictal subgraphs are equally sensitive to connections in the SOZ, or are some interictal subgraphs more sensitive than others. We hypothesized that connectivity in the SOZ would be expressed in a few interacting brain regions, rather than homogenously over many functional subgraphs. Thus, we expected the SOZ sensitivity measure to stratify functional subgraphs based on the epileptic network architecture within and outside the SOZ. First, we separately ranked each patient’s subgraph clusters in decreasing order of their average interictal SOZ sensitivity. Next, we aggregated SOZ sensitivity measures for interictal subgraphs of the same ranked cluster across patients. We expected cluster ranking to reveal potential hetereogeneity in the SOZ sensitivity of interictal subgraphs. Across the patient cohort, we generated a distribution of the average SOZ sensitivity for each of the top 6 ranked clusters, the minimum number of subgraph clusters identified for the 22 patients (Fig. 4*A*). Using a paired $t$-test and Bonferroni correction for multiple comparisons, we compared the SOZ sensitivity distribution of each cluster to a null model in which brain regions within the SOZ are randomly permuted for every interictal subgraph. Compared with the null distribution, we found significantly greater SOZ sensitivity for cluster 1 (μ=0.31±0.04; $t_{21}=8.19,\;p<2\times 10^{-7}$; Table 2, d), cluster 2 (μ=0.15±0.03; $t_{21}=5.58,\;p<3\times 10^{-5}$; Table 2, e), and cluster 3 (μ=0.08±0.02; $t_{21}=3.75$, *p* < 0.005; Table 2, f); significantly lower SOZ sensitivity for cluster 6 (μ=−0.10±0.02; $t_{21}=-3.97$, *p* < 0.001; Table 2, g); and no significant difference for cluster 4 (μ=0.01±0.02; *t*_21_ = 1.86, *p* = 0.08; Table 2, h) and cluster 5 (μ=−0.05±0.02; $t_{21}=-1.47$, *p* = 0.16; Table 2, i). These results indicate interictal subgraphs exhibit a heterogeneous sensitivity to brain regions within and outside the SOZ, with subgraphs in cluster 1 demonstrating the presence of network hubs localized to the SOZ and subgraphs in cluster 6 demonstrating the presence of network hubs localized outside the SOZ (Fig. 4*B*).

**Figure 6.**
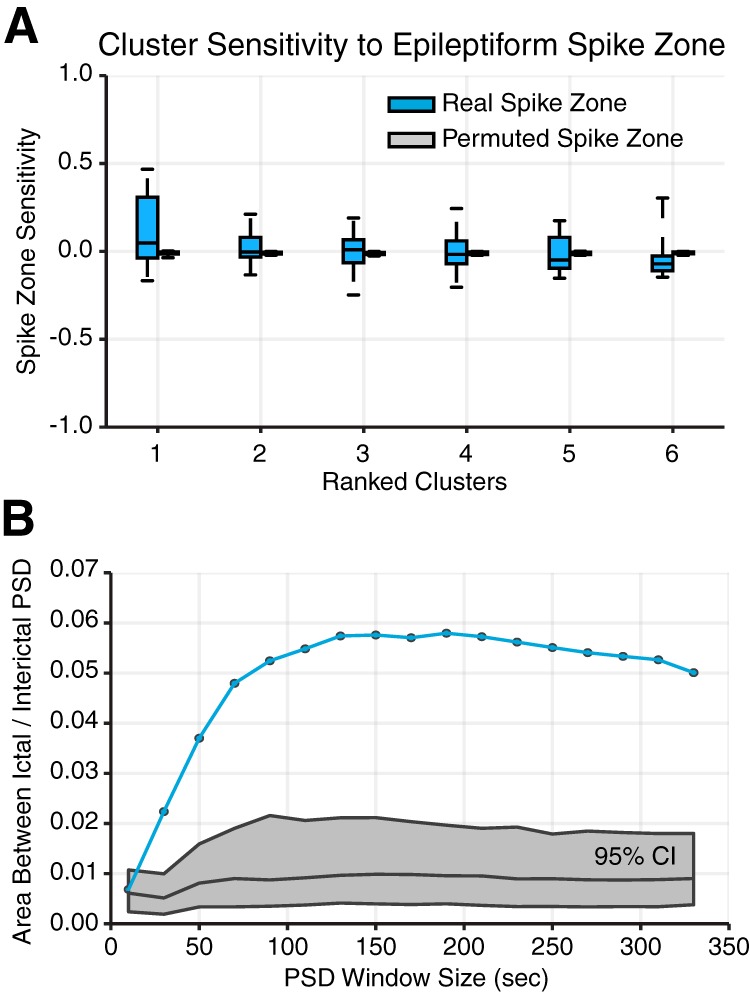
Methodological considerations. ***A***, Distribution of average spike sensitivity of subgraphs in each cluster, from each patient (*N* = 22). Spike sensitivity of true spiking regions in blue and of permuted spiking regions in gray. We observed no significant effect of subgraph cluster assignment on interictal spike sensitivity (one-way ANOVA; *F*_5_ = 1.50, *p* = 0.20). We also found no significant differences between spike sensitivity for real spiking regions compared with permuted spiking regions (paired $t$-test; *t*_21_ < 2.2, *p* > 0.05; Bonferonni corrected for multiple comparisons). These results demonstrate that functional connectivity described by subgraphs is not sensitive to network regions that exhibit interictal spikes. ***B***, Mean area between PSD curves for ictal and interictal subgraphs for different window sizes used in the calculation of the PSD. True area in blue and 95% confidence interval using FDA in gray. These results demonstrate that our finding of differences in subgraph expression dynamics during ictal and interictal epochs is robust to choice in window size used to compute the PSD.

We next asked whether interictal subgraph topologies are uniquely sensitive to architecture in the SOZ, or whether they capture a wider range of interictal epileptiform activity. This question is critical for understanding whether functional connectivity captured by subgraphs is artifactually driven by interictal epileptiform spikes, which do not necessarily correlate with regions within the site of seizure initiation. To determine the degree to which a subgraph expressed functional connectivity in spiking regions, we quantified the relative connection strength between brain regions exhibiting spikes and between brain regions not exhibiting spikes for each subgraph by computing the spike sensitivity measure. First, we computed the average spike sensitivity over subgraphs of each ranked cluster. Next, we generated a patient-level distribution of the average spike sensitivity for each cluster (Fig. 6*A*). To assess whether subgraph clusters differentially capture functional connectivity between spiking regions, we used a one-way ANOVA and found no significant effect between ranked cluster assignment and average spike sensitivity (one-way ANOVA; *F*_(5)_ = 1.50, *p* = 0.20; Table 2, m). Next, we asked whether any particular cluster exhibited subgraphs that were more sensitive to spiking regions than expected by a null model in which brain regions exhibiting epileptic spikes were randomly permuted for every interictal subgraph. Using a paired $t$-test and Bonferroni correction for multiple comparisons, we found no significant differences between the average spike sensitivity of any cluster and its associated null distribution ($t_{21}<2.2$, *p* > 0.05; see Table 2, n--s, for clusters 1-6). These results suggest that interictal subgraph topology is not driven by brain regions that demonstrate abnormal epileptiform spiking.

Taken together, we found that the epileptic network decomposes into a small number of constituent subgraphs that predict varying degree of architecture between brain regions within the SOZ, ranging from strongly connected to significantly disconnected. Moreover, interictal subgraph topology is uniquely sensitive to brain regions in the SOZ and not grossly driven by brain regions that periodically emit epileptic spikes. Overall, the stratification of subgraphs based on sensitivity to the SOZ suggests that different network substructures may be complicit with the SOZ during interictal periods. It follows that relative differences in the temporal expression of functional subgraphs could describe time periods in which distributed network regions are interacting. If subgraphs with high sensitivity to functional connectivity within the SOZ exhibit different temporal patterns of expression than subgraphs with low sensitivity, then such dynamical properties might be useful for predicting SOZ-sensitive subgraphs from interictal epochs.

Accordingly, we next examined how various functional subgraph topologies differentially behave in their pattern of time-varying expression: that is, their subgraph dynamics.

### Temporal dynamics differentiate subgraphs of interictal and ictal epochs

We have presented evidence that ictal subgraphs are topologically similar to interictal subgraphs and, further, that interictal subgraph topology can predict where seizures begin. Logically, we finally ask, if ictal and interictal subgraphs express similar network architecture, how is functional connectivity of the epileptic network differentially expressed between ictal and interictal epochs? By addressing this critical question, we aimed to explain how network architecture involving the SOZ remains active during interictal epochs without manifesting clinical seizures. First, we analyzed the time-varying expression coefficients of each subgraph, which represent the degree to which a subgraph is expressed as a function of time. These coefficients are naturally provided by the NMF subgraph detection technique. From these data, we formulated two hypotheses: (1) that functional subgraphs express a variety of dynamical modes that predict subgraph topologies with heightened sensitivity for epileptic brain regions, and (2) that expression of ictal subgraphs is modulated at slower time scales than interictal subgraphs, supporting the notion that seizures are internally driven processes with coordinated dynamics.

To test our first hypothesis, we used established tools for studying network dynamics (Chai et al., 2017) and computed subgraph expression energy, a measure of overall dynamical activity, and subgraph expression skew, a measure of transient or persistent dynamics, and identified a sample of subgraphs that exhibit high/low energy and transient/persistence dynamics (Fig. 5*A*). Intuitively, the subgraph energy measures the intensity with which a subgraph is expressed over a period of time. Subgraphs with greater expression energy tend to be more dominant in the global network architecture, while subgraphs with lower expression energy resemble more quiescent network processes. Based on our finding that interictal subgraphs are sensitive to functional connectivity within the SOZ, we expected that network processes related to the SOZ might be more quiescent during seizure-free interictal periods, leading to predictably lower expression energy for interictal subgraphs with high SOZ sensitivity (cluster 1) compared with interictal subgraphs with lower SOZ sensitivity (clusters 2-6). Using a paired $t$-test and Bonferroni correction for multiple comparisons, we compared the distribution of expression energy, averaged over all interictal subgraphs of each cluster, across patients to the distribution of expression energy, averaged over all interictal subgraphs outside that cluster, across patients (Fig. 5*B*). We found that interictal subgraphs of cluster 1 exhibit significantly lower expression energy (μ=0.17±0.01) than interictal subgraphs outside of cluster 1 (μ=0.20±0.004; *t*_21_ = –3.21, *p* = 0.004; Table 2, j), suggesting that, indeed, subgraphs with high sensitivity to SOZ brain regions exhibit significantly attenuated activity during interictal epochs. Importantly, our results imply that expression energy is specific in its ability to predict the subgraph cluster that exhibits strong functional connections in the SOZ.

Next, we asked whether interictal subgraphs with pronounced connectivity in the SOZ (cluster 1) differ in their pattern of expression compared with interictal subgraphs with pronounced disconnectivity in the SOZ (cluster 6). To answer this question, we used subgraph expression skew to determine the overall transience of a subgraph. Intuitively, subgraph transience measures the behavior in which a subgraph is expressed over a period of time. Subgraphs with greater transience tend to exhibit intermittent increases in expression and may resemble brief periods of heightened recruitment in conjunction with metabolic demand or cognitive goals, while subgraphs with lower transience exhibit more routine fluctuations and may resemble persistent and essential network processes. During interictal periods, we expected that subgraphs with strong connectivity in the SOZ may express their pattern of functional connections with greater transience, in support of seizure initiation, than subgraphs of cluster 6 with significant disconnectivity in the SOZ (Fig. 5*C*). Using a paired $t$-test we found that expression skew, averaged over all interictal subgraphs, across patients was greater for cluster 1 (μ=1.25±0.24) than cluster 6 (μ=0.75±0.20; *t*_21_ = 2.12, *p* = 0.04; Table 2, k). These results suggest interictal subgraphs with high connectivity within the SOZ are expressed transiently, and interictal subgraphs with high connectivity outside the SOZ are expressed persistently. In other words, network substructures highly involved with seizure-onset areas intermittently increase in expression, while structures highly involved outside the SOZ, and quiet within the SOZ, persist in their expression during interictal periods.

These results point to a robust repertoire of interictal dynamics involving different component subgraphs of the epileptic network and suggest that network regions in the SOZ may be involved in quiescent, low-energy processes that intermittently increase in dominance without manifesting clinical seizures. While findings related to our first hypothesis paint a nuanced picture of the role played by epileptic network architecture in interictal dynamics, answers to our second question regarding how network processes associated with subgraphs differentially evolve during ictal and interictal epochs remain elusive.

Specifically, we sought an understanding of the different time scales associated with network processes during ictal and interictal epochs. To address this issue, we computed PSD for each subgraph, averaged the PSD curves over all ictal or interictal subgraphs of each patient, and analyzed the resulting ictal and interictal PSD distribution (Fig. 5*A*). Using a statistical technique called FDA (see Ramsay and Silverman, 2005, for technique; and Bassett et al., 2012, for illustrative application), we asked whether the area between ictal and interictal PSD curves were significantly different by comparing the true area to a null model in which ictal and interictal labels across subjects were permuted 1,000,000 times, and the area between the curves was recomputed for each permutation. We found that the ictal and interictal PSD curves were significantly separated (area between curves = 0.014; *p* = 2.2 × 10^−5^; Table 2, l), indicating that, on average, network processes underlying ictal dynamics are more likely to operate at frequencies lower than 0.2 Hz, while network processes underlying interictal dynamics are more likely to operate at frequencies >0.2 Hz. To ensure that our finding is not driven by differences in the duration of ictal and interictal epochs, we recomputed the area between PSD curves using several window sizes (Fig. 6*B*) and observed consistently significant separation between ictal and interictal PSD curves for window sizes >10 s (Bonferroni corrected FDA; *p* < 0.05; Table 2, t). Our finding that the expression of ictal subgraphs modulates at slower frequencies and expression of interictal subgraphs modulates at higher frequencies, implies that the same epileptic network architecture of ictal and interictal epochs support network processes at vastly different time scales. More generally, these results demonstrate that seizures mark a critical shift in network dynamics that is driven by slower and more coordinated expression of frequently interacting brain regions.

## Discussion

In this work, we asked the following question: does interictal functional architecture of the epileptic brain perpetuate network dysfunction several hours between seizures? To answer this question, we designed and applied a novel tool to disentangle subgraphs and their time-varying expression from dynamic functional connectivity. Our work supports the notion that ictal and interictal epochs traverse a similar set of functional subgraphs, but differ in the temporal pattern of subgraph expression: that is, subgraph dynamics.

### Subgraphs disentangle regions of the epileptic network

A common notion in epilepsy is that dysfunctional cortical regions produce epileptiform activity, capable of generating seizures. However, network theorists posit that dysfunction may, in part, arise when neural activity between cortical regions hypersynchronize (Uhlhaas and Singer, 2006; Jiruska et al., 2013). Previous studies have identified discrete network states that describe shifts in global network topology, such as magnitude of functional connectivity (Rummel et al., 2013; Burns et al., 2014; Khambhati et al., 2015). However, these approaches are unable to pinpoint specific functional connections that drive changes in brain state across a seizure.

Building on prior work (Eavani et al., 2013; Leonardi et al., 2013, 2014), in this study, we disentangle functional networks into additive subgraphs, patterns of functional interactions between brain regions, that vary in expression over time. Logically, different subgraphs may be simultaneously or sequentially expressed to meet functional demand (Bassett et al., 2006; Deco et al., 2011; Bullmore and Sporns, 2012; Santarnecchi et al., 2014; Chai et al., 2017). Our results demonstrate that the dynamic epileptic network expresses functional subgraphs that recur during ictal and interictal epochs. It is intuitively plausible that the epileptic network is actually composed of a small set of subgraphs that underlie normal function during interictal epochs, but are coopted to support seizure dynamics during ictal epochs (Kramer and Cash, 2012; Schevon et al., 2012; Korzeniewska et al., 2014; Petkov et al., 2014; Khambhati et al., 2015). Such a theory is corroborated by our finding that subgraphs of ictal epochs are more likely to lie at the transition between clusters representing different gross topological architecture, and exhibit slower and more coordinated dynamics than during interictal epochs. The slow subgraph dynamics we observed in ictal epochs operate in a similar frequency range to infra-slow oscillations (0.02-0.2 Hz) of the local field potential, whose putative role is to modulate neuronal excitability (Vanhatalo et al., 2004). Based on this relationship, we speculate that ictal subgraphs may play a mechanistic role in coordinating excitability between brain regions in the epileptic network to drive initiation, evolution, and termination of seizures.

Importantly, the geography of the subgraph projection space points to a core-periphery organization (Borgatti and Everett, 1999) of ictal and interictal subgraphs, in which more densely clustered interictal subgraphs form a core set of highly similar topologies and more loosely clustered ictal subgraphs form a network periphery of more variable topologies. The existence of core-periphery organization in dynamical brain networks related to language (Fedorenko and Thompson-Schill, 2014; Chai et al., 2016b) and learning (Bassett et al., 2013) supports the idea that temporally variable network architectures help navigate different cognitive states. In the epileptic network, ictal subgraphs of the cluster periphery may be more likely to facilitate dynamical transitions between clusters of different subgraph topologies than interictal subgraphs. Furthermore, our finding that subgraphs of seizures with pronounced spatial spread (CP + GTC) lie closer to their cluster periphery than focal seizures (CP) may contribute to global properties of network topology that have been used to predict seizure type in prior work (Khambhati et al., 2016). Neurophysiologically, the epileptic network demonstrates a weakened regulatory, push-pull control in constraining CP + GTC seizures (Khambhati et al., 2016) and might contribute to the ability of CP + GTC subgraphs to more flexibly transition between subgraph clusters than CP subgraphs.

### Predicting seizure origin in the network

We observed that functional interactions specific to the SOZ are highly predicted by the magnitude of functional connectivity and cluster assignments of topologically similar, interictal subgraphs. Our results agree with prior studies demonstrating increased network connectivity in seizure-onset regions during interictal epochs (Warren et al., 2010; Korzeniewska et al., 2014). Our finding that topologically similar subgraphs form clusters over the long data record suggests that the pattern of functional interactions is critical to differentiate regions that drive seizure onset from the surrounding network.

Importantly, our results demonstrate that the site of seizure origin in the epileptic network exhibits dysfunction that recurs transiently over long periods of time. Furthermore, our novel subgraph clustering approach reliably pinpoints this target several hours before seizures occur and reveals that the region is overall more “silent” or dormant relative to regions outside the seizure origin. However, we witnessed that these dysfunctioned and attenuated subgraphs can transiently disrupt functional interactions underlying persistent brain processes not involving the seizure origin. Prior work has shown that focal, left-sided epileptiform activity is associated with decreased short-term verbal memory and focal, right-sided epileptiform activity is associated with decreased short-term memory in nonverbal or spatial tasks (Aarts et al., 1984; Holmes and Lenck-Santini, 2006). Further studies demonstrate that seizures originating in the temporal lobe result in decreased cognitive performance on tasks often associated with activation of frontal and prefrontal lobe, such as performance IQ, verbal IQ, and word list learning (Jokeit et al., 1997), suggesting that cognitive functions are impacted over long distances through network interactions. The approach we developed can be used to study pressing questions regarding secondary deficits caused by interactions between epileptic and nonepileptic brain regions.

### Methodological limitations and extensions

The first important clinical consideration related to this work is the sampling error inherent in any intracranial implantation procedure. Any of the techniques used to map epileptic brain usually yield incomplete representations of the epileptic network. As a consequence, the subgraphs we measured may represent just a portion of more distributed functional circuits that extend further throughout the brain.

Secondly, our methods of predicting epileptic network architecture from interictal epochs rely on accurate delineation of seizure-onset regions. Because of sampling error and variability in clinical decision making, the seizure-onset region may be under- or oversampled. However, the goodness-of-fit of our statistical model in predicting seizure-onset regions based on functional connectivity suggests that our model reasonably agrees with a consensus definition of the SOZ formed by a team of practicing neurologists.

### Clinical impact

Mapping architecture of the epileptic network presents significant challenges for clinicians. In patients with neocortical epilepsy, we showed that functional network topology is highly similar between ictal and interictal epochs. These findings are relevant for (1) optimizing treatment strategies to reduce dysfunction and preserve normal function, and (2) reducing morbidity and mortality associated with extended duration of invasive intracranial electrode implantation, which according to recent studies may actually require months of outpatient intracranial recording with implantable devices (King-Stephens et al., 2015). By predicting seizure-onset regions from interictal epochs, clinical monitoring may be shortened, or potentially even conducted intraoperatively. In this setting, one might imagine epilepsy surgery or device placement taking place in one procedure, relying on interictal brain network mapping, delivered similarly to ablations performed by cardiac electrophysiologists. Furthermore, our finding that complex patterns of functional connectivity correlate with sources of dysfunction supports the use of novel interventional strategies, such as laser ablation or implantable devices, to affect functional circuits at finer spatial scales than is currently possible with large resective surgery.

## Synthesis

Reviewing Editor: Liset Menendez de la Prida, Instituto Cajal CSIC Decisions are customarily a result of the Reviewing Editor and the peer reviewers coming together and discussing their recommendations until a consensus is reached. When revisions are invited, a fact-based synthesis statement explaining their decision and outlining what is needed to prepare a revision will be listed below. The following reviewer(s) agreed to reveal their identity: Michal Zochowski, Levin Kuhlmann

The ms was reviewed by two referees and myself independently. Both reviewers agree the ms advance the field and that you present an interesting methodological approach to investigate ictal and interictal network dynamics in terms of functional connectivity. We discussed however there are several methodological issues that require further revision. Data presentation should be also improved to report results from all patients more clearly in supporting exemplary cases shown in figures. Since there are many details to correct I include below the three independent reports. For us the critical points are:

- To clarify homogeneity of database. If you feel your database will gain more consistency by excluding some patients, please proceed.

- Reorganize ms so that Method section comes first and clarify several methodological concerns listed in the reports.

- Report individual patient data and mean group data more clearly.

- Statistical issues are of major important in eNeuro. Please, carefully consider and describe your statistical design since you ms may require additional review.

We feel you should be able to address all our concerns in less than few months, and therefore my decision is to revise and re-review.

R1-comments:

In this manuscript the authors quantify temporal evolution of functional connectivity in patients having epileptic seizures. Namely they compare evolution of functional connectivity matrices for ictal and interictal periods. They find that functional connectivities during ictal and interictal periods are generally similar, with FC during interictal regions being able to predict seizures onset zones during the seizures.

Assuming that functional connectivity matrix is (to some degree) a correlate of structural network connectivity this result is not extremely surprising. However these results are novel and will be of interest to the community.

I have few questions/comments that I would like authors to address:

1. I was surprised to see that functional connectivity is more stable (i.e. the distance from centroid was smaller) for the interictal periods that for ictal ones. The ictal dynamics seems more internally driven and therefore potentially more stereotypic as compared to externally driven cognitive events in interictal periods. On the other hand authors lump together patients with SP, CP and CP+GTC seizures. Especially the last group could potentially see temporally evolving graphs during different stages of seizures yielding larger distance from the centroid. It might be useful to separate CP seizures and CP+GTC seizures (possibly not enough statistics for other ones) and ran the analysis separately for different types of seizures.

2. The authors do themselves disfavor presenting the examples on Figure 5 using different time scale bars for ictal and interictal events. Also it seems that the amplitudes of parameters for ictal and interictal events have different magnitude (different y-scale). Is this an universal result, i.e., that the magnitude of expression of given sub-graphs was significantly stronger for ictal then interictal regimes? Will this not scew the result? If that is the case that takes me back to previous point about the ictal dynamics being potentially more stereotypic.

3. Technical question: was the number of ictal and interictal epochs internally controlled for every patient?

R2-comments:

The authors present an interesting methodological approach to analysing interictal and ictal network dynamics and highlight there are similarities in the subgraph structure during ictal and interictal periods, and show that the subgraphs appearing in both periods appear transiently in seizures and persistently in interictal periods. This is useful for targeting brain structures and networks for seizure control based on electrical stimulation.

Main comments:

1. The abstract makes it sound like you will consider the links between epilepsy and cognitive networks (i.e. “Prior studies demonstrate focal, neocortical epilepsy is associated with decreased cognitive performance, several hours before seizures” and “Our observations suggest that the epileptic network drives dysfunction by controlling dynamics of functional interactions between brain regions that generate seizures and those that underlie cognitive function”) but doesn't quite get into any details with cognitive networks. That's ok since what is presented is interesting. Maybe more links to cognitive networks can be made by considering where your electrodes are localised in terms of structural MRI atlased brain areas. This would require CT of the electrodes and structural MRI for each subject. Independent of cognition, it would still be interesting to understand any relationships between interictal and ictal subgraphs and their actual spatial location. i.e. are the interictal subgraphs that appear in ictal periods localisable to certain brain areas? Are these remote brain areas, or areas likely to facilitate seizure propagation?

2. You say “interictal subgraph topology can predict where seizures begin”. Is this for a given seizure or just generally at the statistical level for a set of a subject's seizures? E.g. do you have multifocal seizure subjects where a subject has a seizure start in one area at one time and then later another different seizure starts in another area. Will the interictal subgraph topology predict the different locations of these different seizure onsets?

3. Generally its important to make it very explicit what your observations/samples are for a statisical test. Sometimes it is hard to tell if samples represent individual epochs or averages from an individual. This could be made clearer for all statistical tests used. E.g. Figure 5 caption, does the one-way repeated measures anova apply to subject HUP68 or the group? It reads as though it applies to the individual but I would have thought you meant it to apply to the group where the average value from each subject represents a sample. It's obviously more interesting if you say something about the group. If it is applied to the individual data the repeated measures part of the repeated measures ANOVA only deals with the correlation between the ictal and interictal data and not the correlation of the ictal samples, or interictal samples, with themselves (since they are part of a times series), so I don't think this statistical test can be applied. If you take the average skewness value for each subject separately for ictal and interictal data to be the samples contributed from an individual, then you can use the RM ANOVA since you treat the subjects as independent and the only correlation is between their ictal and interictal data.

Other comments:

4. The use of interictal networks for epilepsy diagnosis and treatment has been developed a lot by John Terry's group and probably deserves citing. E.g. this work incorporates human interical functional connectivity into computer simulations of seizure onset:

Petkov, G., Goodfellow, M., Richardson, M. P., & Terry, J. R. (2014). A critical role for network structure in seizure onset: a computational modeling approach. Frontiers in neurology, 5.

5. You say “used the average k from 30 epochs as the representative number of subgraphs to learn from all ictal and interictal epochs of the patient”. Don't you get a single optimal k from one set of 30 epochs? Don't you mean to say “Used the average k, \bar{k}, from K resamplings of 30 epochs as the representative...”.

6. You also need to define \bar{k} more explicitly.

7. What are the values of M and \bar{k} and \bar{j} for each subject?

8. Is there a reference for this part of the methods “we retrieved the cluster assignment with maximum likelihood for each subgraph and counted the number of times each possible pair of subgraphs was assigned to the same cluster { and by extension the probability that any two subgraphs cocluster.”? Otherwise, what you have done is ambiguous.

9. Can you add a column to table 1 listing “number of 5 min interictal epochs”? Number of channels and info on grids, strips and depth arrays could be useful.

10. missing “notch” - Each window was “notch” filtered

11. missing “the” - expression from “the” epileptic brain

12. “We develop new methods to identify clusters of functionally interacting brain regions from 100 hours of intracranial recordings in neocortical epilepsy patients” - Don't you want to say “per patient”? Otherwise it reads like you only use 100 hours of total data. In the results you say you have 105 +/- 17 hours per patient.

13. Can you make it explicitly clear in a single sentence that epochs refer to periods of 5 minutes and windows refer to periods of 1 second?

14. It seems like you only use clinical seizures (i.e. versus clinical equivalent or sub-clinical). Can you make this more explicit?

15. Most of the first 5 paragraphs of the results contains methods and should go in the methods section.

16. What does it mean to have a continuous range of values for the temporal coeffcient of a subgraph? Does high temporal coefficient mean most of a given subgraph is active and a low temporal coefficient mean only part of the subgraph is active?

17. Figure 3, Are the one-way repeated measures tests applied for each individual or once across the group? For some subjects in figure 3D it looks like the ictal data has a shorter normalised distance to centroid.

18. This sentence could be clearer “We analyzed the SOZ sensitivity in subgraphs across our patient cohort by choosing the minimum number of subgraph clusters in all 22 patient, or 6 subgraph clusters”. I thought the minimum number considered was 5 clusters for given patient, or are you talking about minimum optimal j, \bar{j}?

19. For the mixed models can you state more explicitly what the observations/samples are?

20. I prefer to see statistical tests defined in the methods, but if there are word limits I understand.

Editor's comments:

- The work presumably focuses on focal seizures (i.e. clearly identified SOZ), but the some patient exhibit generalized seizures (study 033) and laterality is not clear in other (study 037). Since data were obtained from an open access portal, it may be possible that all details are not provided and homogeneity may be questioned.

- This is a complex paper. Several methodological details are intermingled with results. One has to go back and forth to follow the rationale. Writing may be optimized. Material and Methods section should come before Results. This will facilitate understanding the approach.

- Regarding data stationarity (page 13, line 375): there is strong delta contribution, especially during seizures. This casts doubts regarding stationarity between windows (1 sec non-overlapping, high-pass filtered 1 Hz). This should be carefully considered. What frequency band is more affected? Would results be similar if data is filtered between 4-120 Hz?

- Regarding initial processing and approach:

a) Authors claim they obtain a normalized cross-correlation between each pair but equation 1 does not fit. It is not normalized. The limits of tau are not defined (10%, 20% of the window?). It is not clear what does it mean that xk=yk=0.

b) It is unclear what matrix is used for the NMF method since this should be applied over a bi-dimensional matrix >0 and A is tri-dimensional (because of time; line 389), and it may be negative (equation 1)

c) It is useful to refer to Fig. along Methods to help the reader follow the approach. If I understood well matrices at top right are obtained from equation 1 (after clarifying dimensionality); matrices at bottom right are from equation 2; sub-graphs at bottom left are from equation 3. What is V in eq 3. (Note reference to Fig.S2 and S3 are wrong at line 428).

d) Subgraph clustering requires initializing conditions acting as seeds (lines 106-112). How much dependent are results from this? Please, clarify why are you initializing with a deterministic method to find subgraphs and with a random method to do consensus clustering? Also, how are surrogates designed and tested is unclear (lines 113-117). It may be useful to show all this in Fig.2. Also here, not clear why high similarity (green) fully applies to all within activity subgraphs (interictal and ictal).

- Most of results reflect data from a single patient (HUP68; Fig.4,5). Group data should be reported in all cases.

- Fig.3a,b: How is the Euclidean space defined? What are x and y?

- Fig.5: it seems that different frequency components dominate ictal versus interictal subgraphs. This may bias cluster identification and stationarity issues may be relevant.
